# Influence of COPD systemic environment on the myogenic function of muscle precursor cells *in vitro*

**DOI:** 10.1186/s12931-022-02203-6

**Published:** 2022-10-14

**Authors:** Carme Casadevall, Antonio Sancho-Muñoz, Ignacio Vicente, Sergi Pascual-Guardia, Mireia Admetlló, Joaquim Gea

**Affiliations:** 1 Institut Hospital del Mar d’Investigacions Mèdiques (IMIM), C/ Dr. Aigüader 88, 08003 Barcelona, Spain; 2 Centro de Investigación Biomédica en Red de Enfermedades Respiratorias (CIBERES), 08003 Barcelona, Spain; 3 Department of Medicine and Life Sciences (MELIS), Universitat Pompeu Fabra (UPF), 08003 Barcelona, Spain; 4 Pulmonology Department, Hospital del Mar-IMIM, 08003 Barcelona, Spain; 5 Hospital de l’Esperança, Av. Santuario, Ptge. de Sant Josep la Muntanya 12, 08024 Barcelona, Spain

**Keywords:** COPD systemic environment, Muscle precursor cells, Myogenic capacity, Gene expression

## Abstract

**Background:**

Loss of muscle mass and function are well-recognized systemic manifestations of chronic obstructive pulmonary disease (COPD). Acute exacerbations, in turn, significantly contribute to upgrade these systemic comorbidities. Involvement of myogenic precursors in muscle mass maintenance and recovery is poorly understood. The aim of the present study was to investigate the effects of the vascular systemic environment from stable and exacerbated COPD patients on the myogenic behavior of human muscle precursor cells (MPC) *in vitro*.

**Methods:**

Serum from healthy controls and from stable and exacerbated COPD patients (before and after Methylprednisolone treatment) was used to stimulate human MPC cultures. Proliferation analysis was assessed through BrdU incorporation assays. MPC differentiation was examined through real-time RT-PCR, western blot and immunofluorescence analysis.

**Results:**

Stimulation of MPCs with serum obtained from stable COPD patients did not affect myogenic precursor cell function. The vascular systemic environment during an acute exacerbation exerted a mitotic effect on MPCs without altering myogenic differentiation outcome. After Methylprednisolone treatment of acute exacerbated COPD patients, however, the mitotic effect was further amplified, but it was followed by a deficient differentiation capacity. Moreover, these effects were prevented when cells were co-treated with the glucocorticoid receptor antagonist Mifepristone.

**Conclusion:**

Our findings suggest that MPC capacity is inherently preserved in COPD patients, but is compromised after systemic administration of MP. This finding strengthens the concept that glucocorticoid treatment over the long term can negatively impact myogenic stem cell fate decisions and interfere with muscle mass recovery.

**Supplementary Information:**

The online version contains supplementary material available at 10.1186/s12931-022-02203-6.

## Background

Loss of muscle mass and function, particularly of lower limb muscles, are well-recognized systemic manifestations of chronic obstructive pulmonary disease (COPD) [1]. The mechanisms underlying these changes have a multifactor etiological basis [2–6] and have a negative impact on prognosis and mortality [7–9]. Acute exacerbations, in addition to their impact on pulmonary function, further promote deterioration of skeletal muscle dysfunction [10–13]. COPD Patients who experience frequent acute exacerbations have more severe muscle wasting along with incomplete recoveries that result in a significant loss of muscle mass and performance overtime [10].

Mechanisms leading to muscle wasting have been mainly attributed to an imbalance of anabolic and catabolic processes, resulting in the loss of protein content [14, 15]. However, muscle wasting may be further aggravated by an impaired recovery of skeletal muscle mass due to an imbalance of apoptosis and cellular regeneration and repair [15]. Accordingly, an increased number of apoptotic nuclei have been detected in the quadriceps muscle of both normal weight and underweighted COPD patients [16, 17]. Although the muscle regeneration process appears to be a plausible mechanism to regulate muscle mass [18], studies aimed at analyzing its involvement in normal- and low-weight COPD patients have received limited attention [15; 19, 20].

Reconstruction of the skeletal muscle tissue after damage, repeated exercise, or as a result of disease, is facilitated by the action of satellite cells (SCs), a population of myogenic precursor cells (MPCs) located between the basal lamina and the sarcolemma of myofibers [21]. In adult skeletal muscle, they are normally quiescent, but in response to environmental stimuli they become activated, proliferate extensively, migrate, and fuse to existing myofibers to repair damage and/or facilitate an increase in its size. This orderly progression of events is controlled by transcription factors such as Pax7 and specific muscle regulatory factors including Myf5 and MyoD (expressed at early stages), as well as myogenin and Myf6 that regulate later stages of myogenic differentiation. The formation of multinucleated myotubs expressing muscle structural proteins such as Myosin isoforms and Alpha-actin reflect a complete differentiation and maturation of the contractile skeletal muscle cells [21]. This myogenic process is modulated, at the same time, by diverse inflammatory mediators, such as cytokines and prostaglandins [22, 23].

The SC capacity to support tissue maintenance depends on their abundance and function. SC function, in turn, is a result of the interplay between external cues, arising from local and systemic factors, and the inherent capability of the cells to respond to those cues and undergo myogenesis. Thus, it is likely that alterations in the vascular systemic environment may influence satellite cell behavior affecting their contribution to muscle homeostasis and repair [24, 25].

In stable COPD patients and, particularly during exacerbation episodes, there is a profound modification of the systemic environment including increased levels of inflammatory and oxidative stress mediators which may contribute to muscle impairment [2, 3; 6; 26, 27]. Additionally, systemic inflammatory markers and cytokines such as IL-6, IL-8, tumor necrosis factor alpha (TNF-alpha), fibrinogen, α1-antitrypsin, myeloperoxidase and C-reactive protein (CRP) are further increased during the exacerbation episodes [28]. Moreover, therapeutic strategies with systemic glucocorticoids, despite beneficial clinical benefits, are not devoid of risks [29]. Bearing all this in mind, we hypothesized that circulating factors present in COPD serum may affect myogenic capacity of MPCs and hamper its contribution to recover muscle mass. The aims of the present study were to: (i) investigate the effects of serum from stable and exacerbated COPD patients on human MPC function *in vitro*, and (ii) to elucidate the effects of Glucocorticoid (GC) treatment during acute exacerbations in this cell model.

## Methods

### Study population

A total of 12 subjects participated in the study as donors of muscle precursor cells. Muscle specimens from the *vastus lateralis* were obtained from 4 healthy subjects, 4 COPD patients with preserved weight and 4 COPD patients with low weight (Table 1 A). Lung function was evaluated through determination of spirometric values, static lung volumes and diffusion capacity following standard procedures. COPD was diagnosed according to currently available guidelines [30, 31]. Nutritional status was assessed using different indicators such as Body Mass Index (BMI) and determination of body composition by bioelectrical impedance (Fat Free Mass Index, FFMI) [32]. Low weight was defined as BMI ≤ 20 and/or FFMI ≤ 16 kg/m^2^.

Additionally, 21 subjects were enrolled to supply their blood samples in order to obtain necessary sera for this study: 7 healthy subjects, 7 stable COPD patients (SCOPD) and 7 COPD patients with an acute exacerbation (AECOPD) (Table 1B). Stable COPD patients, defined as patients without any exacerbation of COPD during the previous 3 months were recruited from a hospital outpatient clinic. COPD patients with an acute exacerbation, defined as respiratory symptomatic worsening that led to a change in medication, were recruited upon hospital admission.

The study conforms to the principles outlined in the Declaration of Helsinki and was approved by the ethics committee of our institution (CEIC-IMAS; Ref. 2007/2961/I). Written informed consent was signed by all participants before enrolment.

### Muscle precursor cell culture

Muscle precursor cell (MPC) cultures were established from biopsies of the *vastus lateralis* as previously described [32]. After removal of fat and connective tissue, the muscle specimen was finely minced with sterile scissors, digested with 0.2% collagenase type I (Sigma-Aldrich) for 60 min and 0.05% trypsin (Gibco) for 30 min at 37 °C with occasional agitation. Cells were then pre-plated in a culture dish for 3 h to eliminate undesired cells by plastic adhesion. Unattached cells were removed and seeded on 1.5% gelatin-coated tissue culture dishes for propagation in first passage in proliferation medium (PM: DMEM/M199, 20% FBS and 1% P/S/Fungizone supplemented with insulin, hFGF and hEGF). Cells were grown at 37 °C in a humidified atmosphere of 5% CO_2_ and 95% air, and medium was replaced every 48 h. Once 60–70% confluent, human skeletal muscle cells were split into dishes for expansion. Evaluation of proliferation and differentiation capacity was performed in early cultures (between passages 4 to 6) to ensure consistency. All cultures exhibited a high myogenic purity level (> 94%) as determined by immunostaining for desmin (D33; Thermo Fisher Scientific). Cell viability, as assessed by trypan blue exclusion, was well preserved under all circumstances analyzed (> 95%).

### Human serum for culture media supplementation

Whole blood was collected in evacuated sterile serum tubes, clotted for 30 min at room temperature, centrifuged at 1500 g for 15 min to isolate the serum fraction and stored at -80 °C until needed. An equal volume of serum from each individual within a specific group was pooled to be used for cell culture experiments. Serum pools were designated as *hu S CONTROL* when obtained from control subjects, *hu S SCOPD* when obtained from stable COPD patients and *hu S AECOPD* when obtained from COPD patients with an acute exacerbation. *Hu S AECOPD* was obtained both, at the time of hospital admission, and 90 min after intravenous methylprednisolone (MP) administration (40 mg IV bolus injection). This time was chosen to obtain the maximum plasma concentration of MP (which has a short elimination half-life of 2.3 h) before plasma clearance and intracellular metabolism decreases its availability.

### Experimental setup of cell cultures

Myogenic function of cell cultures was examined using a cell and serum cross-over design. Each of the 12 MPC cultures was induced to proliferate and differentiate by exposing them to each specific group of human serum pools: *hu S CONTROL*, *hu S SCOPD* and *hu S AECOPD* obtained before and after MP treatment. Moreover, each of these conditions was analyzed in the absence or presence of 10 µM of the glucocorticoid receptor (GCR) antagonist Mifepristone (MIF) (Sigma-Aldrich, cat # M8046).

For proliferation studies cells were exposed for 4 days to proliferation medium (PM) containing 10% human serum (*hu S*) from each specific group. Myogenic differentiation was induced at a cell confluence level of ∼90% by replacing PM with differentiation medium (DM: DMEM/M199 containing 2% *hu S*). Experiments on myotubes were carried out after 9 days of differentiation.

### Cell proliferation

The effect of each human serum group on each MPC line proliferation was determined by using the colorimetric BrdU cell proliferation ELISA (Sigma-Aldrich) according to the manufacturers’ instructions.

### Immunofluorescence

Cells were seeded at ∼90% confluence on chamber culture slides. After 24 h they were induced to differentiate for 9 days in DM containing human serum from either stable, exacerbated COPD patients or healthy controls as described above. Differentiated myotubes were fixed with 100% methanol for 10 min at -20 °C and incubated overnight at 4 °C with a primary antibody specific to fast myosin heavy chain protein (MHC) (MY-32, Sigma-Aldrich). Then, cells were labelled with a donkey anti-mouse antibody conjugated with Alexa Fluor 488 (Jackson ImmunoResearch Laboratories Inc) and DAPI was used for nuclei staining. Treated cells were observed using a fluorescence microscope (Olympus BX61) equipped with a digital camera with appropriate filters.

### Western blot assay

Western blotting was performed to determine protein levels of the late myogenic markers myosin heavy chain (MHC) and actin alpha 1 (ACTA1) in myotubes. Total cell protein was isolated 9 days after induction of myoblast differentiation as previously described [32]. Equivalent amounts of protein were fractionated by SDS–polyacrylamide gel electrophoresis, electrotransferred to Immobilon-P PVDF membranes (Millipore), blocked in 2% BSA and incubated overnight at 4 °C with primary antibodies against MHC (A4.1025; Millipore) and ACTA1 (5C5; Sigma-Aldrich). After removing the unbound primary antibodies, membranes were incubated with species-specific horseradish peroxidase-coupled secondary antibodies for 1 h. Antibody-associated protein bands were visualized by enhanced chemiluminescence substrate (ECL, Thermo Fisher Scientific). Equal loading of protein was confirmed by stripping membranes and re-probing with antibodies against the glycolytic enzyme glyceraldehyde-3-phosphate dehydrogenase GAPDH (FL-335; Santa Cruz Biotechnology) and tubulin alpha-1 A (B-5-1-2; Sigma-Aldrich). The ratio of the band intensity of the protein of interest to the loading control proteins was calculated. The mean value of normalized intensities from cells treated with COPD patient’s serum was compared to that of cells treated with control subject’s serum to obtain a percent change.

### Real-time RT-PCR

Total RNA was extracted from cell cultures using TRIzol reagent according to the manufacturer’s protocol (Thermo Fisher Scientific). Single-stranded cDNA was synthesized using the Superscript III reverse transcriptase with an oligo-dT primer (Thermo Fisher Scientific).

PCR reactions were performed with the ABI PRISM 7900HT Sequence Detector System using commercially available predesigned TaqMan gene expression assays (Thermo Fisher Scientific): *MYOG*, Hs01072232_m1; *MHC2A*, Hs00430042_m1; *MHC2X*, Hs00428600_m1 and *ACTA1*, Hs00559403_m1. Gene expression levels were normalized to the housekeeping gene *GAPDH*, (Hs99999905_m1) and relative gene expression analysis was performed using the comparative method 2(-Delta Delta C(T)) [33].

### Statistical analysis

Data are presented as means ± SD. Student’s paired *t*-test was used when comparing the effects of two different human serum pools on myogenic proliferation and differentiation for each MPC culture (normally distributed data). Statistical analysis was performed using SPSS software, version 22.0 (Chicago, IL). A p value < 0.05 was considered statistically significant.

## Results

### Clinical characteristics

Clinical characteristics of the MPC and serum donors are shown in Table 1. All patients had a severe disease according to the GOLD criteria [30, 31].

**Table 1:** Subject characteristics

A. Muscle precursor cell donors			
	**CONTROLS** (n = 4)	**COPD** ***Normal weight*** (n = 4)	**COPD** ***Low weight*** (n = 4)
**Demographics**			
Age, years	64.7 ± 3.6	64.7 ± 3.2	69.5 ± 5.9
Gender (M/F, n)	3/1	2/2	1/3
**Body composition**			
BMI, kg/m^2^	24.4 ± 2.1	26 ± 4.1	17.9 ± 0.6 ^¶¶^
FFMI, kg/m^2^	17 ± 0.9	16.9 ± 2.1	13.6 ± 1.2 ^¶¶^
**Lung Function**			
FEV_1_, % pred	95 ± 9.3	34.7 ± 12.6**	38 ± 19.8**
FEV_1_/FVC, %	84.5 ± 8.7	38.8 ± 13.8*	43 ± 19.6*
RV, % pred	103.7 ± 7.1	190.3 ± 31.2*	209.7 ± 84.2
TLC, % pred	93.2 ± 8	107.3 ± 16.5	118.5 ± 23.6
RV/TLC, %	39.7 ± 4.7	62.9 ± 3.2**	66.2 ± 14.3*
DL_CO_, % pred	94.5 ± 5.3	42.7 ± 25.3	48.2 ± 26.1*
**B. Serum donors**			
	***CONTROLS*** (n = 7)	***SCOPD*** (n = 7)	***AECOPD*** (n = 7)
**Demographics**			
Age, years	61.8 ± 6	67.4 ± 10.5	67.4 ± 10.1
Gender (M/F, n)	1/6	6/1	4/3
**Body composition**			
BMI, kg/m^2^	28.8 ± 3.9	23.8 ± 4.6	25.7 ± 3.7
**Lung Function**			
FEV_1_, % pred	104.3 ± 11.5	43 ± 21.1***	35.2 ± 15.1***
FEV_1_/FVC, %	85.5 ± 3.4	38.3 ± 12.2***	39.4 ± 7.8***

Data represent mean ± SD.

Abbreviations: BMI, body mass index; FFMI, fat-free mass index; FEV_1_, forced expiratory volume in 1 s; FVC, forced vital capacity; RV, residual volume; TLC, total lung capacity; DLco, diffusion capacity for carbon monoxide; *SCOPD*, stable COPD patients; *AECOPD*, COPD patients with an acute exacerbation

(*), (**) and (***) express p < 0.05, p < 0.01 and p < 0.001 compared with control subjects

(^¶¶^) express p < 0.01 compared to normal weight COPD patients

We have analyzed the influence of human serum pools on myoblast proliferation and differentiation using human MPCs obtained from each individual subject. As no significant differences in myogenic cell capacity could be observed between the three groups of MPCs when treated with the same pool of human serum, we have combined the data from all cell populations. Thus, the results are presented as the effect of the different pools of serum as a mean of all MPC cultures (n = 12).

### Effects of human serum from stable and exacerbated COPD patients on cell proliferation capacity

**Fig. 1:**
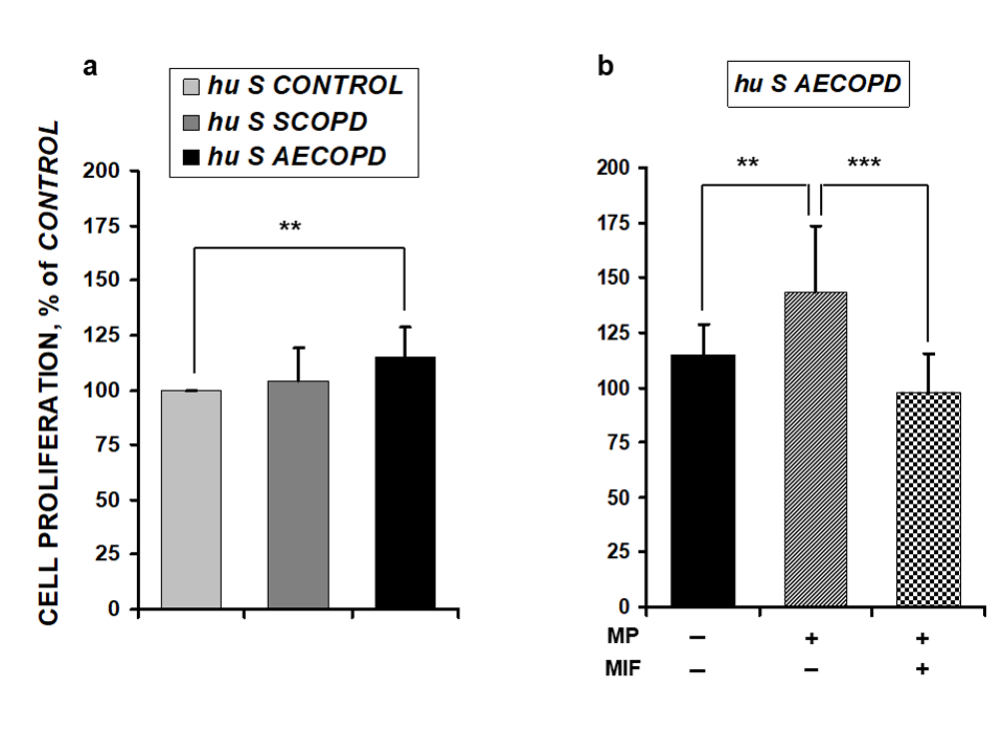
Cell proliferation was assessed by BrdU assay. (a) MPCs were grown in the presence of proliferation medium containing 10% human serum obtained from control subjects (*hu S CONTROL*), stable COPD patients (*hu S COPD*) and COPD patients with an acute exacerbation (*hu S AECOPD*). (b) Proliferation of MPCs in the presence of *hu S AECOPD* serum was assessed before and after methylprednisolone (MP) treatment in the absence or presence of the GCR antagonist Mifepristone (MIF). Results are presented as the mean ± SD (n = 12): **p < 0.01 and ***p < 0.001

MPCs grown in 10% *hu S AECOPD* exhibited a significant increase in cell proliferation compared to cells grown in *hu S CONTROL* while no changes were observed in the presence of *hu S SCOPD* (Fig. 1a).

To determine the effects of MP on myogenic proliferation, MPC cultures were treated with *hu S AECOPD* obtained 90 min after intravenous MP treatment in the absence or presence of the GCR antagonist MIF. Presence of MP in serum induced the highest proliferation rate (Fig. 1b). Simultaneous treatment of these cells with MIF prevented the proliferation increase. On the other hand, treatment with MIF did not elicit any significant effect on MPC cultures exposed to human control or stable COPD serum (data not shown).

### Effects of human serum from stable and exacerbated COPD patients on differentiation capacity

**Fig. 2:**
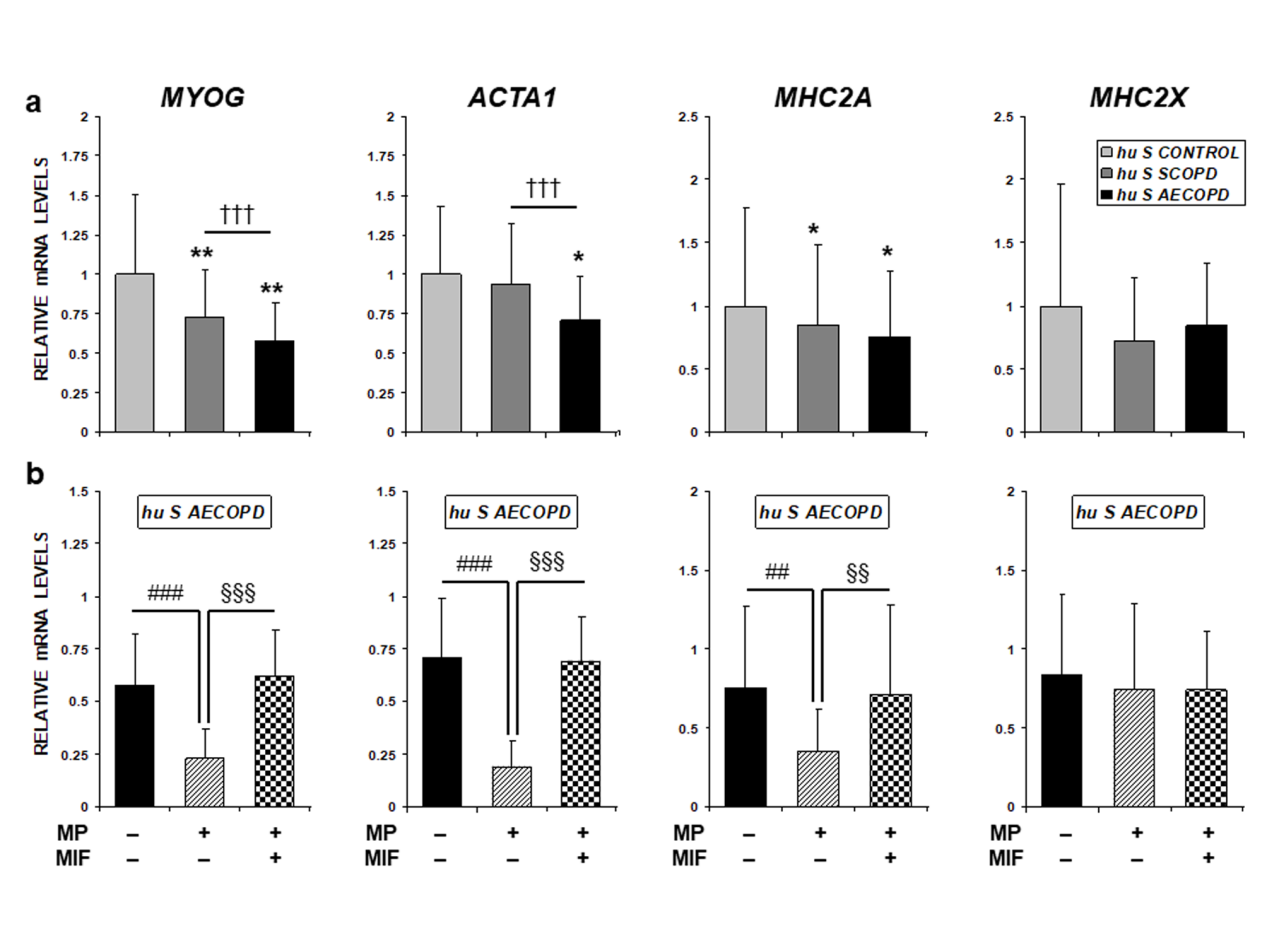
Transcript expression of myogenin (*MYOG*), actin alpha 1 (*ACTA1*) and myosin heavy chain-IIa (*MHC2A*) and -IIx (*MHC2X*) were assessed by real-time RT-PCR. (a) MPCs were induced to differentiate in the presence of differentiation medium containing 2% human serum obtained from control subjects (*hu S CONTROL*), stable COPD patients (*hu S SCOPD*) and COPD patients with an acute exacerbation (*hu S AECOPD*). Results are presented as the mean ± SD (n = 12): *p < 0.05, **p < 0.01 and ***p < 0.001 compared with *hu S CONTROL* group. ^†††^p < 0.001 compared with *hu S SCOPD* group (b) Differentiation of MPCs in the presence of *hu S AECOPD* serum was assessed before and after methylprednisolone (MP) treatment in the absence or presence of the GCR antagonist Mifepristone (MIF). ^##^p < 0.01 and ^###^p < 0.001 compared with *hu S AECOPD* exposure before MP treatment; ^§§^p < 0.01 and ^§§§^p < 0.001 compared with *hu S AECOPD* exposure after MP treatment

All MPC cultures were examined with regard to their ability to differentiate. After 9 days of differentiation, cells were harvested for RNA and protein extraction and gene expression analysis. As shown in Fig. 2a, cells induced to differentiate in the presence of COPD patients’ serum showed reduced mRNA expression of the early differentiation marker *MYOG* and the late markers *ACTA1* and *MHC2A*. *MHC2X* did not show any change. Moreover, *hu S AECOPD* obtained after MP treatment was able to further reduce the expression of these genes (Fig. 2b). Simultaneous treatment of cell cultures with MIF was able to prevent the MP effects (Fig. 2b) but did not induce significant changes in the absence of MP (data not shown).

**Fig. 3:**
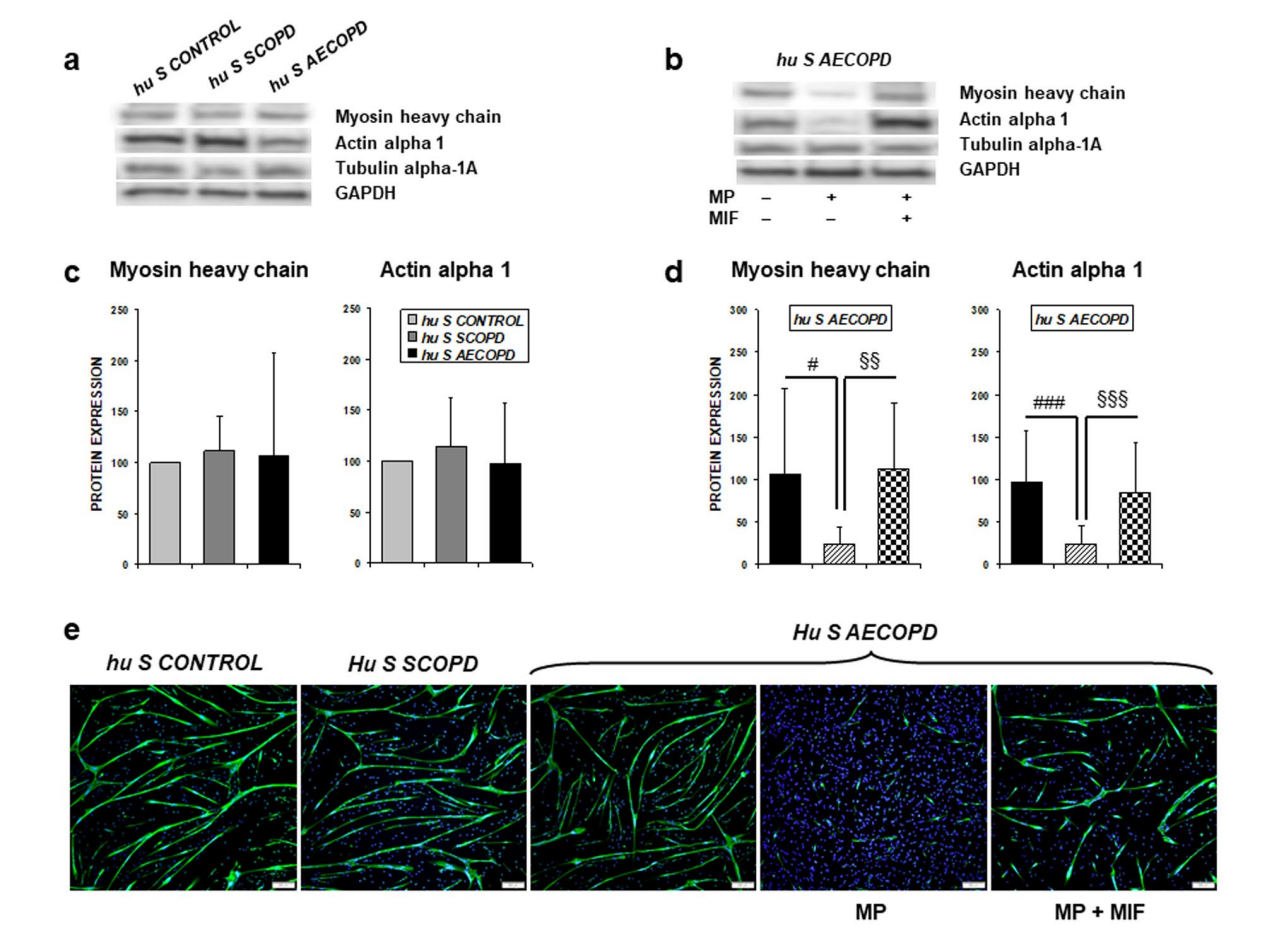
Protein expression and myotube formation. (a-b) Representative western blots. (a, c) MPCs were induced to differentiate in the presence of differentiation medium containing 2% human serum obtained from control subjects (*hu S CONTROL*), stable COPD patients (*hu S SCOPD*) and COPD patients with an acute exacerbation (*hu S AECOPD*). (b, d) Differentiation of MPCs in the presence of *hu S AECOPD* serum was assessed before and after methylprednisolone (MP) treatment in the absence or presence of the GCR antagonist Mifepristone (MIF). Results are presented as the mean ± SD (n = 12). ^#^p < 0.05 and ^##^p < 0.01 compared with *hu S AECOPD* exposure before MP treatment; ^§§^p < 0.01 and ^§§§^p < 0.001 compared with *hu S AECOPD* exposure after MP treatment. (e) Representative microscope images of myotubes monitored by immunofluorescence. Myotubes are stained with an antibody against MHC (green) and nuclei are stained with DAPI (blue). Scale bar = 200 μm

To confirm these results at the protein level, western blot analyses were performed by using antibodies against ACTA1 and MHC proteins (Fig. 3). As opposed to mRNA changes, COPD patients’ serum did not induce significant changes at the protein level (Fig. 3a and c). On the other hand, use of *hu S AECOPD* after MP treatment resulted in a significant reduction of these sarcomeric proteins, while co-treatment of cells with MIF prevented the effects of MP (Fig. 3b and d; Additional file 1: Fig S1).

To further analyze the ability of MPCs to differentiate we performed immunofluorescence studies. In accordance with results obtained by western blot analysis, cells induced to differentiate in the presence of COPD patients’ serum formed roughly a similar number of multinucleated myotubes than cells differentiated in the presence of control serum (Fig. 3e). Here again, presence of MP in *AECOPD* serum was associated with a reduced myotube formation which was also prevented by the presence of MIF.

## Discussion

In the present study we aimed to examine the influence of stable and exacerbated COPD patients’ serum (before and after MP treatment) on human MPC growth and differentiation *in vitro*. We provide evidence that proliferation was increased in the presence of serum obtained from *AECOPD* patients and was further induced after MP treatment. During the differentiation phase, while COPD patients’ serum was able to transcriptionally downregulate most myogenic markers, no significant changes were observed with their protein levels. Treatment with MP, on the other hand, negatively affected MPC differentiation as indicated by reduced expression of differentiation markers and a decreased myonuclear accretion.

Muscle atrophy is a devastating manifestation that frequently occurs in chronic inflammatory disorders such as COPD [34], and substantially contributes to morbidity and mortality [1–3; 7–9]. This deleterious process is largely driven by excessive protein breakdown and is influenced by multiple physical, genetic and molecular factors including inactivity, systemic inflammation, oxidative stress or steroid treatment [2–4; 6]. Acute exacerbations of the disease further contribute to worsen disease related drivers which, in turn, aggravate muscle wasting and dysfunction [10–13].

Skeletal muscle homeostasis and maintenance depends on an intricate balance between protein metabolism and myonuclear turnover. While it is widely recognized that excessive protein degradation is the major process leading to COPD muscle wasting, little is known regarding the effects of systemic inflammation and repetitive exacerbations on myogenic stem cell function [35]. To address this question, we developed an *in vitro* system with human MPC cultures to determine the influence of serum from stable and exacerbated COPD patients with and without MP treatment.

The myogenic potential of MPCs isolated from control subjects or COPD patients (either with a preserved or low weight) did not show any significant differences when exposed to the same human serum group. These results support the absence of intrinsic cellular differences attributable to the COPD condition. As opposed to our findings, a decreased regenerative capacity of MPCs obtained from COPD patients has been previously reported [19]. The reasons for this discrepancy are unclear but could be related to methodological considerations (serum used in both studies comes from different animal species). Further sources of difference could be accounted by intrinsic heterogeneity between human biopsy samples and small numbers of myogenic cultures analyzed.

Gene expression changes underlying muscle stem cell fate decisions, however, were modified when exposed to different serum groups, supporting the notion that systemic factors present in COPD patients may modulate myogenic stem cell biology. MPCs proliferated and differentiated to a similar extent when exposed to serum from control subjects and stable COPD patients. This indicates that myogenic stem cells were able to respond properly to disturbed systemic environment as a consequence of the chronic low-grade systemic inflammation present in stable COPD patients.

The more pronounced systemic inflammation present in serum from AECOPD patients elicited a mitotic effect by increasing myogenic cell proliferation. Indeed, some inflammatory markers that are known to possess a mitogenic effect on MPCs such as IL-6, IL1-beta or TNF-alpha [36] have been reported to be higher in plasma from patients with AECOPD compared with patients with stable COPD and healthy controls [22]. However, the systemic factors modulating MPC functioning, and the exact mechanisms remain to be characterized.

Differentiation induction in presence of this mitotic systemic environment resulted in a transcriptional downregulation of *MYOG* (a muscle regulatory factor that acts as a master regulator of myogenic differentiation) and the late differentiation markers *ACTA1* and *MHC2A*, but not *MHC2X*. In our study, the use of an antibody that labels all Myosin isoforms in western blot analyses could account for the divergence with *MHC2A* transcript levels. However, taken together, these transcriptional changes did not translate to the protein level and did not affect the progression of the myogenic program toward the formation of multinucleated myotubes. RNA and protein expression changes are highly dynamic in mammalian cells and their levels are controlled by multitude of posttranscriptional mechanisms that may account for discrepancies in their concentration [37]. In our system, changes at the RNA level induced by *AECOPD* systemic environment were counteracted at the protein level and the differentiation outcome was successfully achieved.

AECOPD serum obtained after MP treatment, on the other hand, elicited important changes along the myogenic process. The effects of excess GCs on muscle mass have been well characterized [38]. The underlying mechanisms include stimulation of catabolic processes and inhibition of protein synthesis. Although reduced myogenic capacity is another important determinant of skeletal muscle wasting, the effects of GC on myogenesis and muscle mass recovery have received limited attention. In addition, studies conducted on GC-effects on myogenic function have raised conflicting results. Our present findings show that the mitotic effect exerted by *AECOPD* serum on MPCs was further increased when cells were exposed to serum containing MP. These findings are in line with *in vitro* studies in which low concentrations dexamethasone (DEX) increased proliferation rate of primary myoblasts dose dependently [39]. However, other *in vitro* studies, have demonstrated inhibitory effect of DEX on C2C12 proliferation (a mouse skeletal muscle cell line) [40–42].

The increased proliferative rate, however, was not followed by an adequate differentiation of MPCs. Presence of MP in the *AECOPD* serum had a negative impact on the myogenic program downregulating the expression of the differentiation markers (*MYOG*, *ACTA1* and *MHC2A*) both, at the RNA and protein level. These molecular changes resulted in a reduced myoblast accretion and myotube formation as verified by fluorescence immunolabeling. Although a few studies have reported a pro-differentiation effect of GCs on different cell lines [43–45], our findings agree with most reports showing inhibitory effects of GC on myogenesis [42; 46–48]. Concomitant addition of the GCR antagonist MIF to the myogenic cultures exposed to *AECOPD* serum containing MP prevented proliferation and differentiation effects indicating that MP-induced changes are largely mediated by GCRs.

The studies referenced above, delineating GC effects *in vitro*, primarily experimented with the immortal C2C12 mouse myoblast cell line, rather than a finite human myogenic cell line as used in our experimental methods. Thus, differences in the behavior of mouse versus human myoblasts, finite versus immortalized cell line, or a cell line specific effect, could account for these disagreements. Although murine models sometimes can translate directly to human conditions, it has been shown that murine myogenesis differs substantially from human myogenesis [49]. Moreover, finite cultures are more suitable than immortalized cell lines for investigating *in vivo* skeletal muscle growth and differentiation as they represent a model that is closer to *in vivo* situations and clinical applications. Furthermore, the cellular effects of GCs *in vitro* vary with both the origin of the cells (species) and with the nature and concentration of the steroid employed. Therefore, differences in the GC type and concentration used may also underlie the conflicting data.

One limitation of this study is linked to serum pooling. While pooled serum samples provide an effective way for multiple testing when MPCs availability is limited, there could be individual outliers in the pool that could account for the effects detected. Another drawback of this study is that of standard cell culture techniques since they do not mimic physiological conditions. Culturing of MPCs in a controlled environment does not consider the constant complex interactions that take place into the skeletal muscle microenvironment. Thus, additional *in vivo* studies will be needed to further determine the significance of our results.

## Conclusion

Our findings suggest that MPC capacity is not compromised in COPD patients neither in stable conditions nor during exacerbations. MP treatment, on the other hand, resulted in a reduced efficiency of myogenic differentiation. This finding strengthens the concept that GC treatment over the long term, mainly in frequently exacerbator COPD patients, may translate into reduced effectiveness of satellite cell function which, in turn, may contribute to hamper restoration of muscle wasting.

## Electronic supplementary material

Below is the link to the electronic supplementary material

Supplementary Material 1

## Data Availability

The data that support the findings of this study are available from the corresponding author on reasonable request.

## Abbreviations

COPD: Chronic Obstructive Pulmonary Disease
SCOPD: Stable COPD.
AECOPD: Acute exacerbated COPD
GOLD: Global Initiative for COPD
BMI: Body Mass Index
FFMI: Fat-Free Mass Index
MPC: Muscle precursor cells
SC: Satellite cells
PM: Proliferation medium.
DM: Differentiation medium
hu S: human serum
MP: Methylprednisolone
GC: Glucocorticoids.
GCR: Glucocorticoid receptor
MIF: Mifepristone
Real-time RT-PCR: Real-time reverse transcription polymerase chain reaction
MHC: Myosin heavy chain
MHCIIA: Myosin heavy chain IIa
MHCIIX: Myosin heavy chain IIx
ACTA1: Actin alpha 1
MYOG: myogenin.
GAPDH: Glyceraldehyde-3-phosphate dehydrogenase
Mrna: messenger RNA
