# Supplementary material for: Influence of COPD systemic environment on the myogenic function of muscle precursor cells in vitro
Source: Respir Res. 2022 Oct 14;23:282. doi: 10.1186/s12931-022-02203-6 (PMC9569059; doi:10.1186/s12931-022-02203-6)
Supplement: Supplementary file 1 — Supplementary Material 1 [file 12931_2022_2203_MOESM1_ESM.docx]

**SUPPLEMENTAL MATERIAL**

**INFLUENCE OF COPD SYSTEMIC ENVIRONMENT ON THE MYOGENIC FUNCTION OF MUSCLE PRECURSOR CELLS *IN VITRO***

**Carme Casadevall^1,2,3^, Antoni Sancho-Muñoz^2^,^4^, Ignacio Vicente^5^, Sergi Pascual-Guardia^1,3,4^, Mireia Admetlló^2,3,4^, Joaquim Gea^1,2,3,4^**

^1^ Institut Hospital del Mar d’Investigacions Mèdiques (IMIM), Parc de Recerca Biomèdica de Barcelona (PRBB), C/ Dr. Aigüader 88, 08003 Barcelona, Spain.

^2^ Centro de Investigación Biomédica en Red de Enfermedades Respiratorias (CIBERES), 08003 Barcelona, Spain

^3^ Experimental Sciences Department (CEXS), Universitat Pompeu Fabra (UPF), 08003 Barcelona, Spain

^4^ Pulmonology Department, Hospital del Mar-IMIM, 08003 Barcelona, Spain

^5^ Hospital de l’Esperança, Av. Santuario, Ptge. de Sant Josep la Muntanya 12, 08024 Barcelona, Spain

***Correspondence to:** Carme Casadevall

IMIM (Institut Hospital del Mar d’Investigacions Mèdiques)

PRBB (Parc de Recerca Biomèdica de Barcelona)

C/ Dr. Aigüader 88, 08003, Barcelona, Spain.

Telef: (+34) 933160387

E-mail: [ccasadevall@imim.es](mailto:ccasadevall@imim.es)

**Figure S1.** Full length western blot images


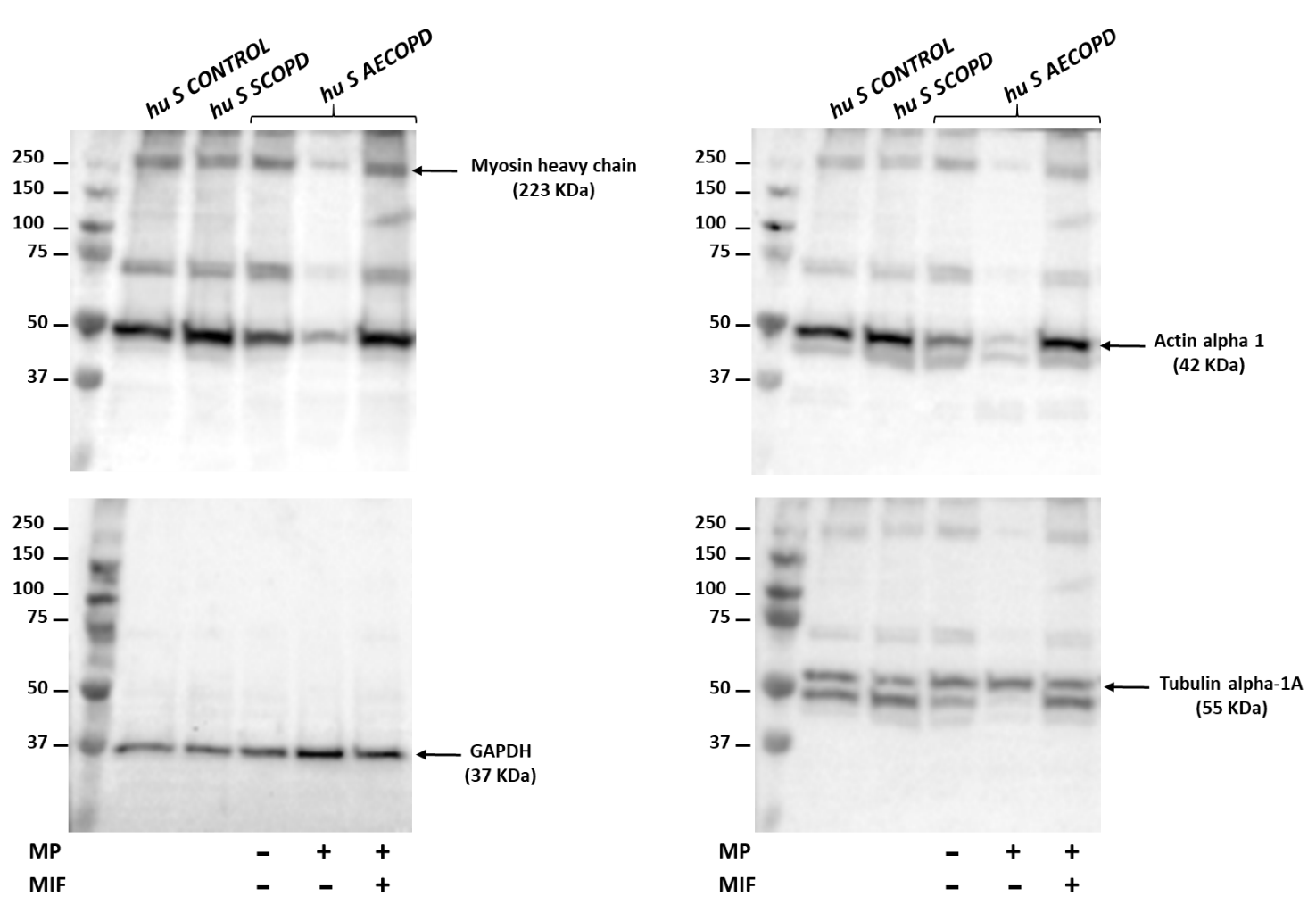


Full length blot images for western blots presented in figure 3a-3d.

MPCs were induced to differentiate in the presence of differentiation medium containing 2% human serum obtained from control subjects (*hu S CONTROL*), stable COPD patients (*hu S SCOPD*) and COPD patients with an acute exacerbation (*hu S AECOPD*). Differentiation of MPCs in the presence of *hu S AECOPD* serum was assessed before and after methylprednisolone (MP) treatment in the absence or presence of the GCR antagonist Mifepristone (MIF).
